# Optimal ROI setting on the anatomically normalized I-123 FP-CIT images using high-resolution SPECT

**DOI:** 10.1007/s12149-016-1107-6

**Published:** 2016-07-20

**Authors:** Masanari Nonokuma, Yasuo Kuwabara, Kosuke Hida, Tomonobu Tani, Koichi Takano, Kengo Yoshimitsu

**Affiliations:** Department of Radiology, Fukuoka University Hospital, 7-45-1 Nanakuma, Jonan-ku, Fukuoka, 814-0180 Japan

**Keywords:** I-123 FP-CIT, SPECT, Specific binding ratio, Statistical parametric mapping, ROI

## Abstract

**Objective:**

The aim of this study is to establish the optimal regions of interest (ROIs) in anatomically normalized I-123 FP-CIT SPECT images for the quantification of dopamine transporter binding.

**Methods:**

The subjects comprised 16 normal controls and 14 Parkinsonian patients. All of the normal control subjects underwent I-123 FP-CIT SPECT and MRI. The SPECT device used in this study was a Toshiba GCA-9300R with triple head detectors. I-123 FP-CIT (148 MBq) was intravenously administered as a bolus, and the SPECT scan started 4 h after the administration. The data were collected over 20 min for each subject, and reconstructed using a 3D-OSEM algorithm. The data were analyzed using SPM8. I-123 FP-CIT SPECT images were anatomically normalized to the MNI space using an I-123 FP-CIT template, and then divided by the background counts automatically measured using the ROIs set for the cerebral cortices.

**Results:**

In the normal control subjects, the specific binding ratios of the MRI-based ROIs were lowest in the caudate nucleus, while the ratios of the I-123 FP-CIT-based ROIs were almost the same throughout all three parts. In contrast, in Parkinsonian patients, the specific binding ratios of the I-123 FP-CIT-based ROIs revealed rostrocaudal decline, while those of the MRI-based ROIs were highest in the anterior putamen.

**Conclusion:**

We created an ROI template on the anatomically normalized MRI and I-123 FP-CIT images, and concluded that I-123 FP-CIT-based ROIs are more suitable for obtaining quantitative values than MRI-based ones.

## Introduction

Quantification of striatal dopamine transporter binding is very important for the evaluation of the presynaptic dopaminergic function in Parkinsonian patients. Several techniques for gathering quantitative information from I-123 FP-CIT (I-123 Ioflupan, DaTSCAN®) SPECT images have been reported [1]. Among them, setting the regions of interest (ROIs) on the anatomically normalized I-123 FP-CIT SPECT images is believed to be useful for the objective and reproducible evaluation of striatal dopamine transporter binding [2, 3]. Also, it is well known that the presynaptic dopaminergic function is known to be more severely impaired in the putamen than that in the caudate nucleus [4]. We, therefore, attempted to create new MRI and I-123 FP-CIT-based ROIs for evaluating the caudate nucleus, anterior putamen, and posterior putamen separately on anatomically normalized brain images, and compared the results between them.

## Subjects and methods

**Fig. 1:**
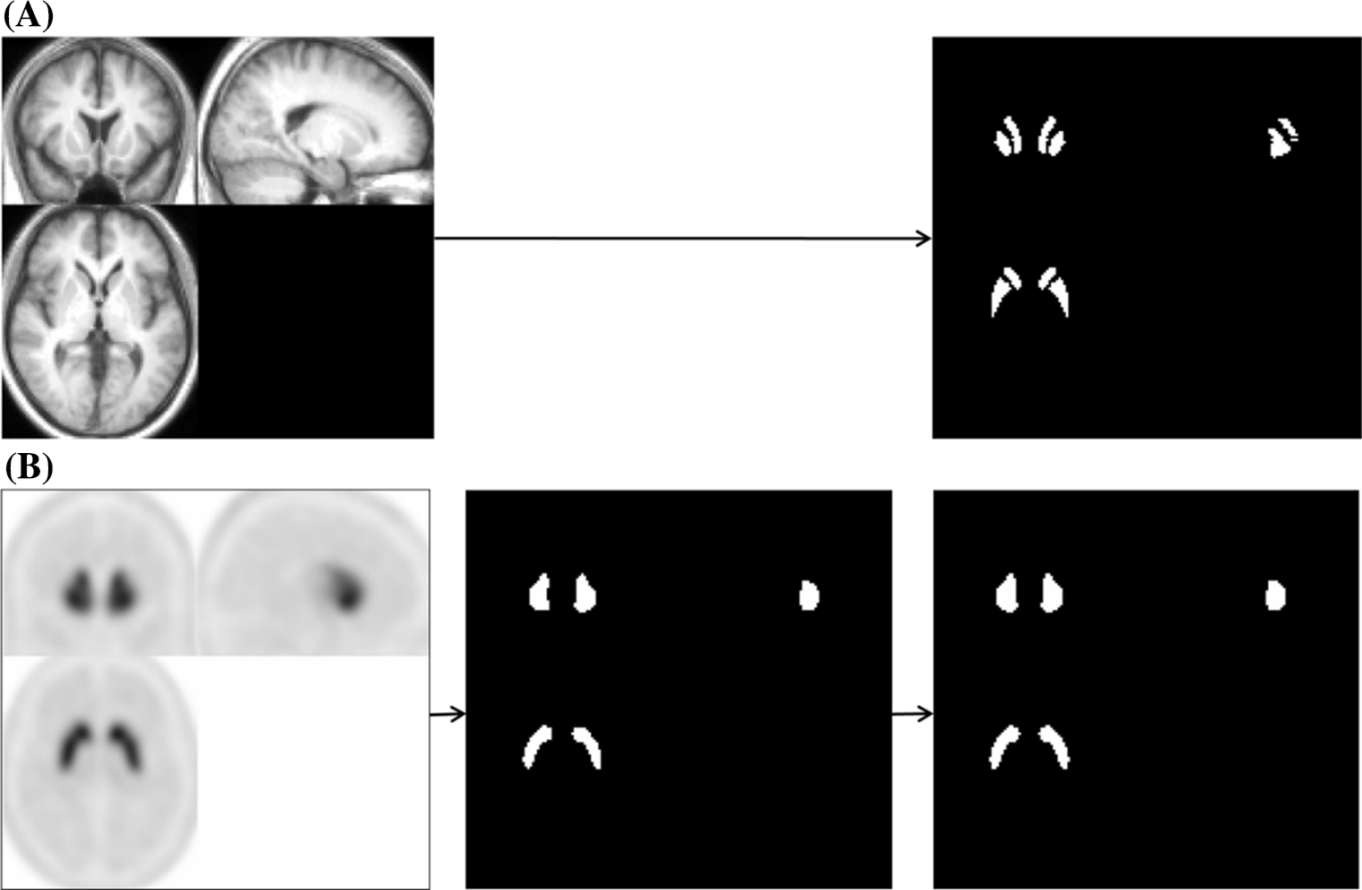
Methods of creating MRI-based ROIs (*upper row*: **a**) and I-123 FP-CIT-based ROIs (*lower row*: **b**). **a** MRI-based ROIs were set manually on the right striatum, and then mirrored. **b** I-123 FP-CIT SPECT-based ROIs were created via the automatic extraction of the striatal region at various cut-off levels. The ROIs were then fused with the reversed ROIs to ensure symmetry

The subjects comprised 16 normal controls (5 females and 11 males; mean age ± standard deviation, 51.6 ± 9.5 years, ranging from 25 to 62 years) and 14 Parkinsonian patients (3 females and 11 males; mean age ± standard deviation, 68.4 ± 10.3 years, ranging from 42 to 83 years). The Parkinsonian patients were classified into stage I (three patients), stage II (five patients), stage III (four patients), stage IV (one patient) and stage V (one patient) by the Hoehn and Yahr scale. All of the normal control subjects underwent MRI. The SPECT device used in this study was a Toshiba GCA-9300R with triple head detectors, which has a spatial resolution of 9.0 mm with fan-beam collimeter in clinical use. I-123 FP-CIT (148 MBq) was intravenously administered as a bolus, and a SPECT scan was started 4 h after the administration. Data were collected for 20 min for each subject, and the I-123 FP-CIT images were reconstructed using a 3D-OSEM algorithm, iteration 4 and subset 15. Attenuation correction was performed using Chang’s method with a μ value of 0.06, while scatter correction was not done. MRI studies were performed via the three-dimensional T1-weighted fast field echo (3D-T1FFE) method using an Intera Achieva (1.5T, Phillips Co. Ltd.) or via the three-dimensional inversion recovery prepared fast spoiled gradient recalled acquisition in the steady state (3D-IR-FSPGR) method using a Discovery MR750w (3T, GE Co. Ltd.), and MRI images of the sagittal plane were obtained in a 240-mm field of view, with a 256 × 256 matrix and 0.75 × 0.94 × 0.94 m^3^ voxel size, and then reconstructed in the axial plane. The data were analyzed using the Statistical Parametric Mapping 8 (SPM8) software program (Wellcome Department of Imaging Neuroscience, Institute of Neurology, University College London). Initially, the I-123 FP-CIT SPECT images of the 16 normal control subjects were co-registered to MRI images, and re-sliced MRI images were normalized to Montreal Neurological Institute (MNI) space using grey.nii template. Co-registered I-123 FP-CIT SPECT images were normalized using the predetermined normalization parameters for MRI images. The anatomically normalized I-123 FP-CIT SPECT images were then divided by background counts individually measured by ROIs set on the cerebral cortices, and averaged I-123 FP-CIT SPECT images of the 16 normal control subjects were created. These averaged I-123 FP-CIT SPECT images were used as a template for the anatomical normalization of I-123 FP-CIT SPECT images of the Parkinsonian patients. Averaged MRI images of the 16 normal control subjects were also created. I-123 FP-CIT SPECT images of the Parkinsonian patients were anatomically normalized to MNI space using I-123 FP-CIT template, and then divided by the background counts automatically measured by the ROIs set on the cerebral cortices. The details regarding the method for creating the I-123 FP-CIT template have been described in our previous study [5]. Three-dimensional ROIs were created with the MRIcro software program, version 1.40 build 1 (http://www.mricro.com; Chris Rorden, Columbia, SC, USA), on both averaged MRI and I-123 FP-CIT SPECT images of normal control subjects after anatomical normalization. MRI-based ROIs were set on the right caudate nucleus and putamen by free-hand on T1WI MRI images and output as ROI images of analyze format. The putaminal ROIs were divided then into the parts: the anterior putamen and posterior putamen. The borders of the anterior and posterior putamen were determined manually as, the area of the anterior and posterior borders of the putamen from the center were almost equal on each axial image. The ROIs on the right side were mirrored and applied to the left side (Fig. 1a). I-123 FP-CIT SPECT-based ROIs were created by extracting the striatal region from the averaged I-123 FP-CIT SPECT images at various cut-off values for the striatal-to-background ratio (3.0, 3.5, 4.0, 4.5, 5.0), and output as ROI images of analyze format. The ROI images were then reversed, and the original images were fused with the reversed images to ensure symmetry (Fig. 1b). The striatal ROIs were divided into the three parts based on both the MRI images and I-123 FP-CIT images. Initially, the borders of the caudate nucleus and putamen were determined by referencing the averaged images of MRI and I-123 FP-CIT of normal control subjects and Parkinsonian patients, and then, the borders of the anterior and posterior putamen were determined manually, as the distances between the anterior and posterior borders of the putamen from the center were almost equal on each axial image. The caudate ROIs were made consciously made larger than the anatomical ones and shifted to the caudal portion. All of the ROIs images were displayed using the FineSRT system developed by Takeuchi et al. [6] to clarify their location in the MNI space. The specific-to-background ratio (S/BG ratio) was obtained by applying these ROIs to each normalized S/BG ratio images. The averaged S/BG ratios on the ROIs of all images for the volume of interest (VOI) were obtained, and then, the values on both sides were averaged. The study protocol was approved by the Independent Ethics committee/institution review board of our hospital.

## Results

**Fig. 2:**
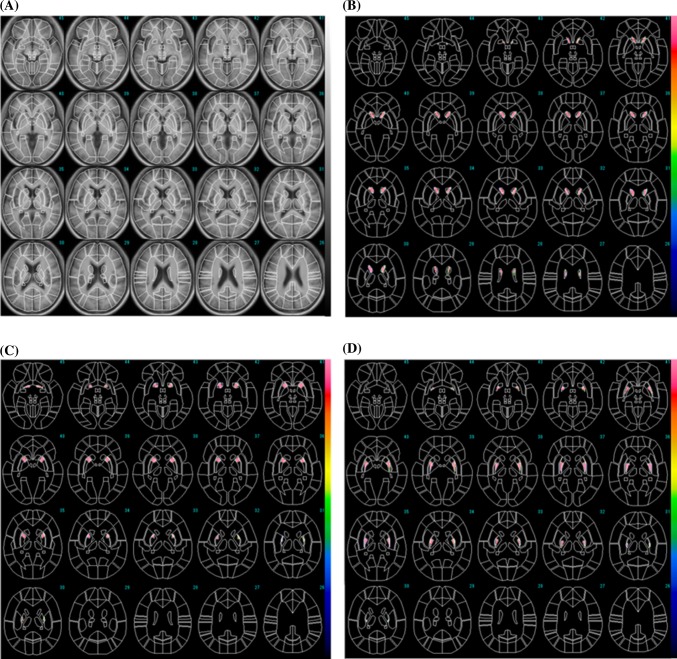
MRI T1-WI images and MRI-based ROIs were shown on the FineSRT system. **a** T1-WI images, **b** caudate ROIs, **c** anterior putamen ROIs, and **d** posterior putamen ROIs displayed using the FineSRT system

**Fig. 3:**
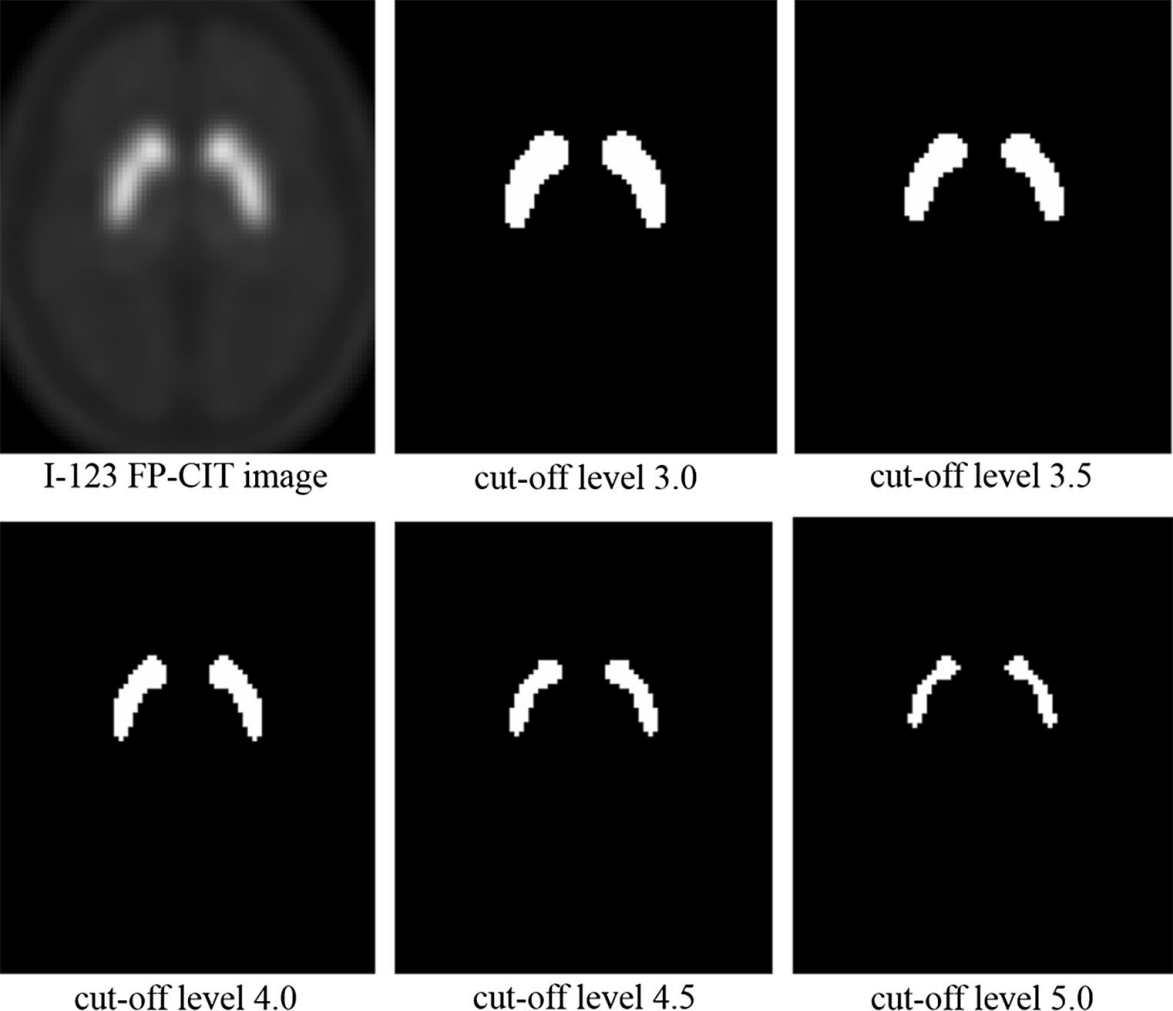
I-123 FP-CIT-based ROIs at various cut-off values

**Fig. 4:**
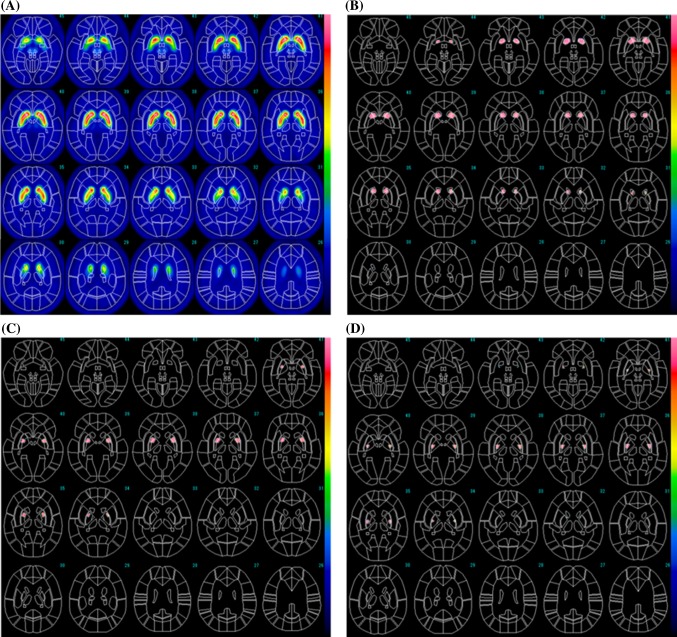
I-123-averaged FP-CIT images of normal control subjects (**a**) and I-123 FP-CIT-based ROIs (**b** caudate nucleus, **c** anterior putamen, **d** posterior putamen) displayed using the FineSRT system

**Fig. 5:**
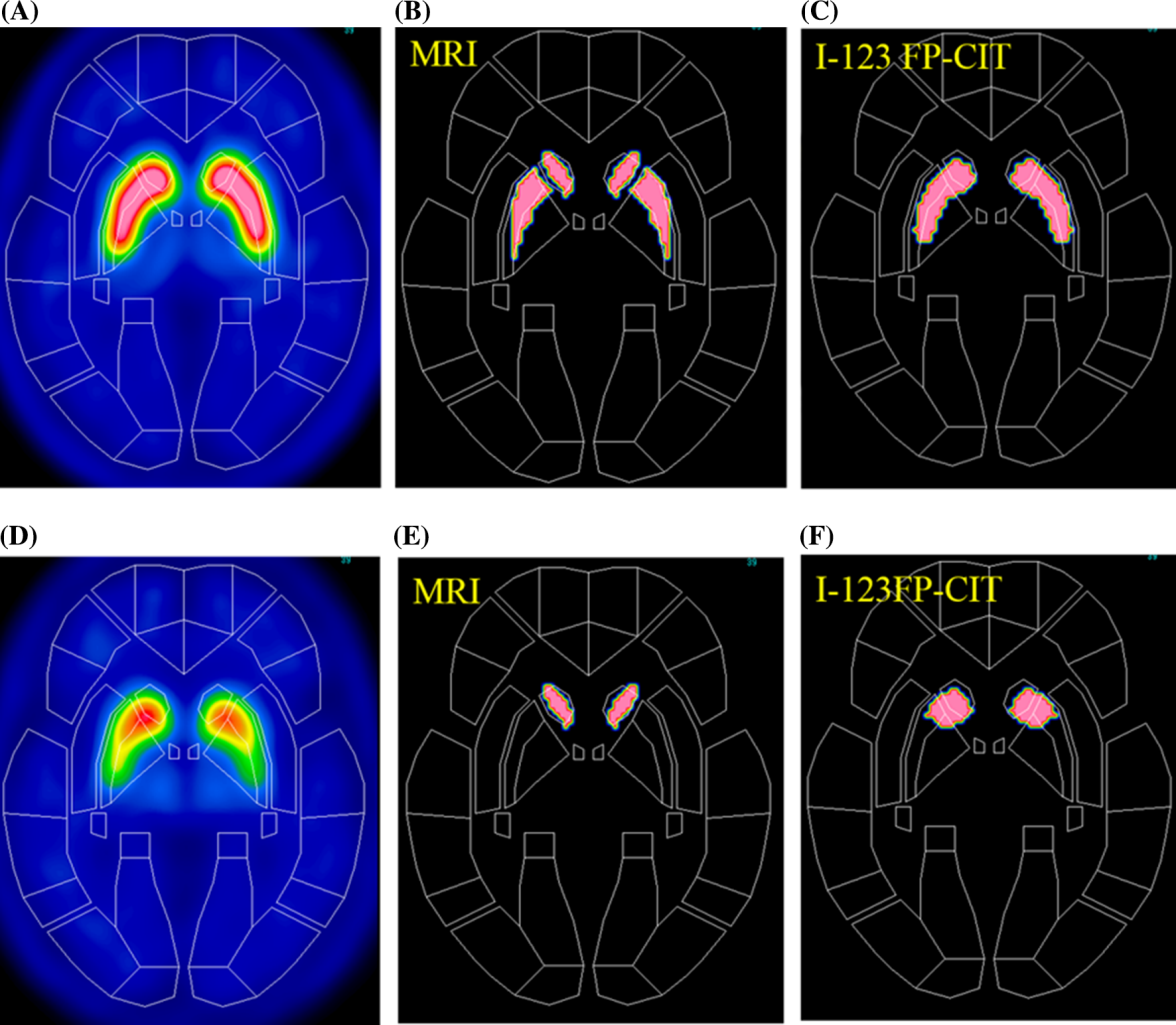
Magnified images of normal control subjects (**a**) and Parkinsonian patients (**d**) displayed using the FineSRT system: **b**, **e** MRI-based ROI, **c**, **f** I-123 FP-CIT-based ROI

**Fig. 6:**
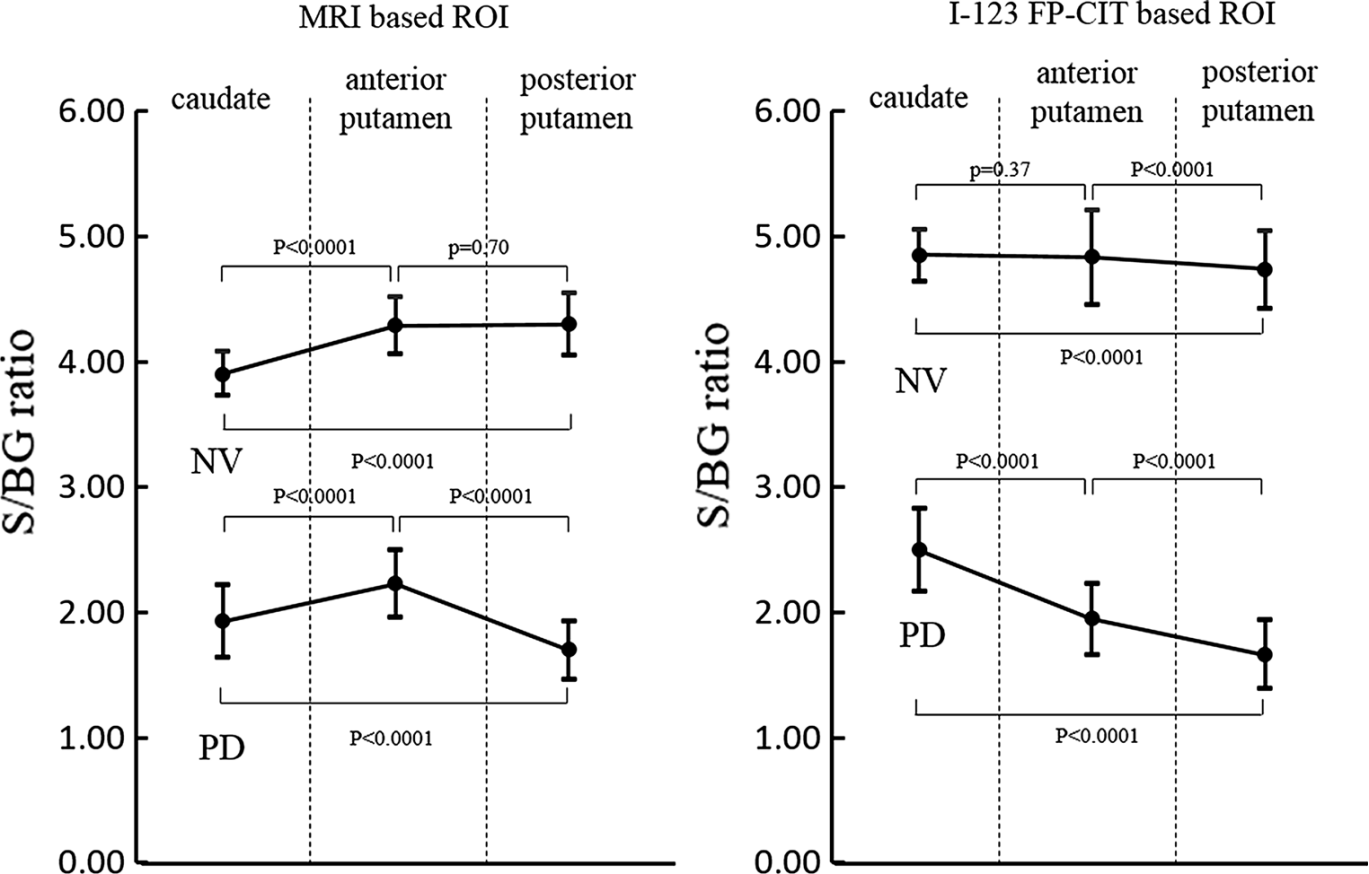
The specific binding ratios (S/BG ratios) of the caudate, anterior putamen, and posterior putamen obtained using the MRI-based ROIs (*left* side) and I-123 FP-CIT-based ROIs (*right* side)

The MRI-based ROIs are shown with T1WI images in Fig. 2 displayed on the FineSRT system. The striatal ROI size was 2122 voxels including 804 voxels for the caudate and 1318 voxels for the putamen (anterior putamen 680 voxels, posterior putamen 638 voxels). The ROIs were well fit to the FineSRT ROI template. The I-123 FP-CIT SPECT-based ROIs at various cut-off levels are shown in Fig. 3. In the visual evaluation, the striatal ROI at the cut-off level of 4.0 was found to be the most similar to the averaged I-123 FP-CIT SPECT image. The ROI sizes were 3220, 2345, 1622, 1075 and 602 voxels at the cut-off levels of 3.0, 3.5, 4.0, 4.5 and 5.0, respectively. The I-123 FP-CIT SPECT-based ROIs are shown with the averaged I-123 FP-CIT SPECT images in Fig. 4 as displayed on the FineSRT system. The striatal ROI size was 1586 voxels including 956 voxels for the caudate and 630 voxels for the putamen (anterior putamen 372 voxels, posterior putamen 258 voxels). Figure 5 shows the magnified images of normal control subjects (Fig. 5a, upper row) and patients with Parkinson’s disease (Fig. 5d, lower row). The averaged I-123 FP-CIT image, MRI-based ROI, and 123-I FP-CIT-based ROI were relatively consistent in normal controls, while the peak radioactivity of the caudate nucleus shifted to the caudal portion of the striatum in patients with Parkinson’s disease. I-123 FP-CIT-based ROIs showed a better fit to the caudate nucleus than MRI-based ROIs in patients with Parkinson’s disease. The averaged ROI values and the standard deviation of the VOI in each part (caudate, anterior putamen and posterior putamen) of the striatum are shown in Fig. 6. In the normal control subjects, the S/BG ratios of the MRI-based ROIs were significantly lower in the caudate nucleus than in the anterior or posterior putamen (*p* < 0.0001, Wilcoxon test), while the S/BG ratios of the I-123 FP-CIT ROIs were not significantly different between the caudate nucleus and anterior putamen (*p* = 0.37). In the Parkinsonian patients, the S/BG ratios of the MRI-based ROIs were significantly higher in the anterior putamen than in the caudate nucleus or posterior putamen (*p* < 0.0001), while the S/BG ratios of the I-123 FP-CIT-based ROIs in the caudate were significantly higher than in the anterior or posterior putamen (*p* < 0.0001), and they also showed a rostrocaudal decline.

## Discussion

In the present study, we created template ROIs for the anatomically normalized I-123 FP-CIT images. The quality of this type of ROI system depends on the performance of the software for anatomical normalization, as the striatum is a relatively small structure and located deep in the brain. SPM is one of the most popular and reliable software programs for statistical imaging analyses [7]. We, therefore, selected SPM8 for the co-registration and special normalization of the images. We first created MRI-based ROIs using MRIcro which is an auxiliary software program for SPM. As shown in Fig. 2, the MRI-based ROI images of the striatum were well fit to the FineSRT ROI template [6]. However, the MRI-based ROIs (Fig. 2) could not accurately catch the radioactivity of the head of the caudate nucleus as shown in Fig. 4, especially in patients with Parkinson’s disease (Fig. 5d). The magnified images of the striatum revealed that the caudate nucleus was rounded on I-123 FP-CIT SPECT (Fig. 5f), in contrast to the discoid shape on MRI (Fig. 5e). This phenomenon is due to the limited resolution of the SPECT device and distortion of the image due to the reconstruction algorithm. We used a high-resolution triple head SPECT device with a fan-beam collimeter, but the spatial resolution was lower than that of MRI or PET. Under these conditions, the tissue mixture effect due to the dilated anterior horn of the lateral ventricle may decrease the radioactivity in the caudate nucleus and shift the peak caudally. Distortion of the images may also be involved in the mismatch between the anatomical location and radioactivity distribution. We, therefore, newly made I-123 FP-CIT SPECT-based ROIs at various cut-off values of the striatal-to-background ratio. Given that the size of the striatal ROI decreases with increasing cut-off value, and that the size of the striatum was deemed adequate at a value of 4.0 on visual inspection, we set 4.0 as the cut-off level for the striatal ROI. The FP-CIT-based ROI size was 1622 voxels, which is 0.76 times of MRI-based-ROIs. The striatal ROI was then divided into three parts: caudate nucleus, anterior putamen, and posterior putamen. We designed the posterior margin of the caudate ROI to shift in the posterior direction, because the radioactivity peak shifted to the caudal portion as shown in Fig. 5f. As a result, the rostrocaudal pattern is more clearly demonstrated by I-123 FP-CIT SPECT-based ROIs than MRI-based ROIs (Fig. 6).

Several studies have attempted to create template ROIs for evaluating anatomically normalized I-123 FP-CIT images. Koch et al. [8] created the I-123 FP-CIT template and 3D ROI map for healthy controls using Brain Analysis Software program (BRASS), which can evaluate the caudate nucleus and putamen separately. Borros et al. [9] created the I-123 FP-CIT template using SPM and SPECT data of patients with drug-induced Parkinsonism, and registered all of the I-123 FP-CIT SPECT images to the new template. These normalized images were quantitatively evaluated using MRI-based VOIs for the caudate nucleus and putamen. Takada et al. [10] applied VOIClassic in Neurostat, a software program for the statistical image analysis of the brain developed by Minoshima et al. [11], to the semi-quantitative assessment of I-123 FP-CIT, and calculated the specific (striatal) to nonspecific (occipital) count ratios on the anatomically normalized images. However, those authors did not describe the difference between the MRI-based and FP-CIT-based ROIs, and the putaminal I-123 FP-CIT uptake was not evaluated separately as anterior and posterior portions. DaTQUANT is a software program supplied commercially by GE Healthcare [12] that can automatically measure the S/BG ratio of the caudate nucleus, anterior putamen and posterior putamen. This ROI system is similar to our ROI system, and seems to be well designed, but we have no experience in using it, and the details of the normalization technique are not clear.

The partial volume effect due to the limited spatial resolution of SPECT device is known to result in underestimation of the ROI values. Bolt et al. [13] reported a method using large ROIs including the striatum to avoid the partial volume effect. Their method involves correction of the total specific radioactivity by the standard striatal volume, and the values obtained by their method are higher than those obtained by conventional ROI methods covering only the striatum. However, this method may underestimate the specific binding ratio, when the striatal ROI includes cerebrospinal fluid space due to cerebral atrophy.

In the present study, we did not try to correct for the partial volume effect, but such correction is believed to be necessary to obtain an accurate value. Several methods other than Bolt’s method seemed useful for partial volume correction. In the future, we will attempt partial volume correction using segmented MRI images [14].

## Conclusion

We created an ROI template on the anatomically normalized MRI and I-123 FP-CIT images, and concluded that I-123 FP-CIT-based ROIs are more suitable for obtaining quantitative values than MRI-based ROIs.
